# RETRACTION: Long Non‐Coding RNA FEZF1‐AS1 Induced Progression of Ovarian Cancer via Regulating miR‐130a‐5p/SOX4 Axis

**DOI:** 10.1111/jcmm.71043

**Published:** 2026-01-28

## Abstract

**RETRACTION**: SunZ. , GaoS. , XuanL. , and LiuX. , “Long Non‐Coding RNA FEZF1‐AS1 Induced Progression of Ovarian Cancer via Regulating miR‐130a‐5p/SOX4 Axis,” Journal of Cellular and Molecular Medicine 24, no. 7 (2020): 4275–4285. 10.1111/jcmm.15088.32135030 PMC7171310

The above article, published online on 05 March 2020 in Wiley Online Library (wileyonlinelibrary.com), has been retracted by agreement between the journal Editor‐in‐Chief, Stefan Constantinescu; and John Wiley & Sons Ltd. A third party reported multiple instances of image duplications between this article and previously published articles. All images in Figure 2C had previously been published in [Zhu et al. 2019 (https://doi.org/10.1038/s41467‐023‐41612‐z)] and reported as different samples. All images in Figures 3A, 3B, 4C, and 4D had previously been published in Li et al. 2017 [(https://doi.org/10.1038/cddis.2017.119)] and reported as different samples. The GAPDH band in Figure 5D and all bands in Figure 5H had previously been published in [Liu et al. 2017 (https://doi.org/10.1186/s12943‐017‐0625‐8)] and all those images were manipulated and rotated. Furthermore, multiple images in Figure 5E had previously been published in Liu et al. 2017, where further manipulation and rotation had been applied to these images.

An investigation by the publisher confirmed these concerns and discovered additional instances of image duplication from other articles. Data in Figure 2D had previously been published in [Liang et al. 2018 (https://doi.org/10.1038/s41419‐018‐0582‐1)], [Wang et al. 2019 (https://doi.org/10.18632/aging.102081)] and [Liang et al. 2020 (https://doi.org/10.1186/s12943‐020‐01206‐5)]. Images in Figure 2C had previously been published in [Zhu et al. 2019 (https://doi.org/10.1038/s41467‐018‐07998‐x)] and these images had been rotated. Multiple images in Figure 5E that were also previously published in Liu et al. 2017, were also later published in [Li et al. 2021 (https://doi.org/10.2147/OTT.S302800)].

The retraction has been agreed to because the evidence of image re‐use and manipulation with multiple previously published articles fundamentally compromises the editors’ confidence in the results presented. The authors did not respond to our notice regarding the retraction.

**RETRACTION**: Z. Sun , S. Gao , L. Xuan , and X. Liu , “Long Non‐Coding RNA FEZF1‐AS1 Induced Progression of Ovarian Cancer via Regulating miR‐130a‐5p/SOX4 Axis,” Journal of Cellular and Molecular Medicine 24, no. 7 (2020): 4275–4285. 10.1111/jcmm.15088.32135030 PMC7171310

The above article, published online on 05 March 2020 in Wiley Online Library (wileyonlinelibrary.com), has been retracted by agreement between the journal Editor‐in‐Chief, Stefan Constantinescu; and John Wiley & Sons Ltd. A third party reported multiple instances of image duplications between this article and previously published articles. All images in Figure 2C had previously been published in [Zhu et al. 2019 (https://doi.org/10.1038/s41467‐023‐41612‐z)] and reported as different samples. All images in Figures 3A, 3B, 4C, and 4D had previously been published in Li et al. 2017 [(https://doi.org/10.1038/cddis.2017.119)] and reported as different samples. The GAPDH band in Figure 5D and all bands in Figure 5H had previously been published in [Liu et al. 2017 (https://doi.org/10.1186/s12943‐017‐0625‐8)] and all those images were manipulated and rotated. Furthermore, multiple images in Figure 5E had previously been published in Liu et al. 2017, where further manipulation and rotation had been applied to these images.

An investigation by the publisher confirmed these concerns and discovered additional instances of image duplication from other articles. Data in Figure 2D had previously been published in [Liang et al. 2018 (https://doi.org/10.1038/s41419‐018‐0582‐1)], [Wang et al. 2019 (https://doi.org/10.18632/aging.102081)] and [Liang et al. 2020 (https://doi.org/10.1186/s12943‐020‐01206‐5)]. Images in Figure 2C had previously been published in [Zhu et al. 2019 (https://doi.org/10.1038/s41467‐018‐07998‐x)] and these images had been rotated. Multiple images in Figure 5E that were also previously published in Liu et al. 2017, were also later published in [Li et al. 2021 (https://doi.org/10.2147/OTT.S302800)].

The retraction has been agreed to because the evidence of image re‐use and manipulation with multiple previously published articles fundamentally compromises the editors’ confidence in the results presented. The authors did not respond to our notice regarding the retraction.

